# The Impact of Designing Near-Peer Teacher Training According to Merrill’s First Principles of Instruction

**DOI:** 10.1007/s40670-024-02267-7

**Published:** 2024-12-27

**Authors:** Kristopher Vaudrey, Marc James Thor Uy, Alexis Thompson, Hannah Morris, Lauren Cifuentes

**Affiliations:** 1 Department of Biomedical Sciences, , Rocky Vista University – Montana College of Osteopathic Medicine, Billings, MT USA; 2 Burrell College of Osteopathic Medicine, Las Cruces, NM USA; 3 New Mexico State University, Las Cruces, NM USA

**Keywords:** First Principles of Instruction, Near-peer teacher training, Near-peer teaching, Medical education, Ultrasound education, Merrill’s First Principles of Instruction

## Abstract

The aim of this study was to assess the effectiveness of an online asynchronous training program for near-peer teachers at the medical school level, designed using Merrill’s First Principles of Instruction. A qualitative phenomenological study using semi-structured interviews and a reflective journal was conducted with second-year medical students (*n* = 10) who participated in the near-peer teaching program for the ultrasound component of the human anatomy lab. Deductive analysis revealed that near-peer teachers benefited. These themes emerged (1) near-peer teachers teaching in the Ultrasound Lab and in future medical practice, (2) activating prior learned knowledge, (3) using videos to help prepare near-peer teachers for teaching in the Ultrasound Lab, (4) providing a self-assessment of content comprehension, and (5) helping near-peer teachers to develop teaching techniques. Future research could evaluate asynchronous training for near-peer teaching in other lab-based activities, explore the impacts of near-peer teaching on medical students who receive near-peer instruction, and determine the longitudinal impacts of such programs.

## Introduction

With the increasing use of ultrasound imaging in clinical practice, medical schools are struggling to find qualified faculty to teach the necessary techniques [1–4]. Smith et al. [3] stated that this lack of qualified faculty presents a training gap in instruction in medical schools, which impacts future residents and doctors in the care of patients. To tackle this challenge, one potential solution is to utilize near-peer teachers (NPTs) who can provide instruction in areas such as ultrasound [3–6].

Near-peer teaching refers to a teaching environment where a more experienced student aids another student [7]. Near-peer teaching is an effective method in medical education that can solve the problem of a shortage of qualified teachers. It benefits both the teachers and students in the educational process and helps in teaching specific skills required for future medical practice [1, 3, 6]. According to Guerrero-Mendivil et al. [8], medical students frequently have the chance to participate in near-peer teaching in medical school. Research often focuses on the impact of instruction on medical students, near-peer teachers, and curriculum but has not delved into the training of near-pear teachers [8]. Upon review of the available literature, further research is necessary to explore features of effective teacher training for near peers in medical education.

NPT training in medical schools is often unstructured, raising concerns about the near-peer teachers’ level of preparation [8]. The limited research demonstrates that creating a formal, structured learning environment focusing on needs-based educational skills might enhance the ability of the near-peer teacher to meet challenges associated with instruction compared to those with little or no formalized training [8–11].

Exploring various methods for training near-peer teachers is essential to address the existing research gap. One promising approach is asynchronous instruction, which can be systematically designed to meet the academic demands of the medical school environment. This format allows medical students to learn at their own pace, free from time and location constraints—particularly beneficial for those with busy schedules [12–14, 24].

In this study, we implemented Merrill’s First Principles of Instruction (FPI) to design two online asynchronous training modules for second-year osteopathic medical students acting as near-peer teachers in the ultrasound component of the Human Anatomy Lab. We chose Merrill’s FPI as our theoretical framework due to its strong research foundation, structured approach, and adaptability for developing a range of instructional materials and experiences. According to Merrill’s principles, effective learning is facilitated through five key strategies: (1) engaging learners in solving real-world problems, (2) activating prior knowledge to support new information, (3) demonstrating new concepts, (4) encouraging the application of this knowledge, and (5) integrating new insights into the learner's existing knowledge base.

The research questions guiding this study are twofold: What aspects of asynchronous training were effective and efficient for near-peer teaching of ultrasound imaging to medical students? Additionally, how significant were Merrill’s five principles in enhancing the design and effectiveness of the asynchronous training modules?

## Methods

### Research Design

In order to address the research questions, this study utilized a qualitative phenomenological research design, allowing for a comprehensive understanding of the effectiveness and efficiency of asynchronous training for NPTs. The phenomenological research design explored the collective significance of individual participant’s experiences of the phenomenon [15]. The data collection consisted of NPT’s semi-structured interviews after completing the two ultrasound labs. The focus of the collected data was on the impact of Merrill’s five principles on the participant’s experiences with the asynchronous training and the impact the training had on their teaching in the HAL course, specifically in the ultrasound component. The researcher documented NPTs’ participation and interactions in each ultrasound lab through a reflective journal containing descriptions of the lab environment and interactions with OMS-1 students. [15].

### Study Personnel

The lead researcher was an instructor in the Department of Anatomy and Cell Biology and served as a co-course director for the HAL course at a southwestern osteopathic medical school where the study participants are students in their second year of medical school. The previous year, the lead researcher was an instructor for the participants in the ultrasound and gross anatomy labs. By having provided instruction in the ultrasound lab, the lead designer/researcher understands how the environment works and the challenges the participants face.

It is important for researchers to acknowledge their own biases when participating in qualitative research [15]. The lead researcher for this study participated in the recruitment, design of the intervention, and data analysis within the study. Therefore, the interviews were conducted by research team members with qualitative interview experience to prevent potential biases resulting from the lead researcher’s relationship with the NPTs.

#### Participant Recruitment

The researcher used convenience and criterion sampling to select participants who could contribute valuable insight to answer the research questions. Convenience sampling involves selecting participants for a sample based on their availability and willingness to participate [16]. According to Creswell and Poth [15], criterion sampling involves selecting participants with previous experience related to the task or phenomenon being studied and meeting specific criteria*.* The criterion sampling included applicants who had to apply to the NPT program, be in good academic standing by the end of their first year of medical school, and have successfully completed the HAL course during the previous academic year. Ten near-peer teachers (four male, six female) were purposefully selected from a cohort of 194 s-year medical students (OMS-2, matriculated in 2022), with all ten successfully completing the study.

During an in-person meeting at the institution’s campus, participants received an institutional review board-approved consent form explaining the study’s purpose, given an opportunity to ask questions, and provide consent to participate. All NPTs who teach the HAL course’s gross anatomy or ultrasound components receive financial compensation from the institution. The participants in the research study did not receive any additional compensation for their online, asynchronous training time.

### Design of the Near-Peer Teacher Training

David Merrill’s [17] FPI design theory framed the intervention’s design. Merrill [17] formulated the set of principles for instructional design by synthesizing various other instructional design theories and models. These principles hold under various conditions, regardless of the methods or models used to implement them [17].

**Fig. 1:**
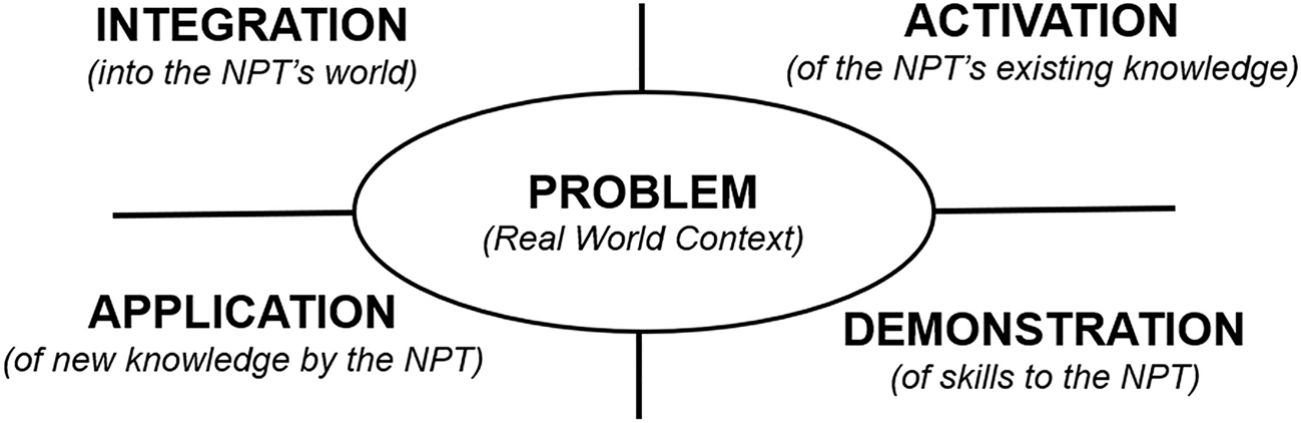
Modified figure—phases for effective instruction [17]

For the NPT training, the designer/researcher applied a systematic cycle of instructional phases based on these principles that focus on real-world problems as the foundation for learning [17]. Merrill’s principles indicate that an instructional designer must explicitly design for problem-solving, activation in the learner, demonstration of key skills, application of those skills, and integration of skills under novel conditions. Figure 1 shows the instructional phases surrounding the centralized real-world or problem-centered task.

#### First Principles of Instruction in the Design Intervention

The designer/researcher explicitly addressed each of Merrill’s principles when developing the NPT training:

1. *Engage learners in solving real-world problems*: In the online, asynchronous training, the designer/researcher provided the NPT with the real-world tasks of ultrasound techniques and image acquisition, teaching advanced ultrasound skills, and addressing scenarios that may present themselves in the lab or in medical practice where the future doctor may need to explain the process to a patient, as well as developing soft skills, such as communication skills and problem-solving to utilize in their future practice [18, 19].
2. *Activate existing knowledge as a foundation for new knowledge*: The instructional design tapped into the NPTs existing knowledge and experiences from the previous academic year’s ultrasound lab that they participated in as students to build a foundation for new knowledge. The designer/researcher used online, asynchronous training modules with PowerPoint presentations that included anatomical imaging reviews, embedded short video links of ultrasound imaging, and refreshers on basic ultrasound techniques to activate the NPTs prior knowledge. This established a strong foundation for learning new skills [17].

The design started with basic tasks, such as anatomical structures that can be seen in ultrasound imaging and basic ultrasound techniques. The complexity of content increased with the progression of the modules, so learners could build on what they were learning in the labs. Within the PowerPoint presentation, the researcher/designer added key teaching tips for ultrasound imaging and the skills to properly communicate the ultrasound techniques to scaffold upon the NPT’s prior foundational knowledge.

3. *Demonstrate new knowledge to learners*: The researcher/designer created content through demonstrations that aligned with the desired learning outcomes, such as identifying anatomical structures and their relationships as seen in ultrasound imaging for NPT training. The online, asynchronous training featured brief demonstration videos that presented ultrasound images and explained the anatomical structures visible in them. Additionally, photographic images were included to clearly illustrate proper patient positioning, ultrasound probe techniques, and image acquisition. The researcher/designer ensured that the media supported effective learning when designing and creating these demonstrations. This included taking advantage of both video and still images and clear and concise text. Krumm et al. [20] emphasized the importance of avoiding materials that increase cognitive load without aligning with the demonstration objectives, specifically regarding the identification of anatomical structures and their relationships in ultrasound imaging, as well as patient positioning.
4. *Encourage learners to apply new knowledge*: The NPTs applied their newly acquired skills and self-assessed their abilities. The objective was to assess the NPT’s knowledge of the newly acquired skills they needed to teach ultrasound imaging. Instruction and self-assessments contained scenarios and questions covering ultrasound techniques, imaging anatomy, the relevant anatomy, patient position, knobology and probe orientation, image acquisition, teaching tips for the upcoming ultrasound lab, and common questions that arise during lab sessions. After each self-assessment, instructors provided feedback. This allowed for guidance on areas that needed strengthening and filled gaps in the NPT’s knowledge. If the NPTs had further questions, they could email or phone the research team members.
5. *Integrate new knowledge into the learner’s world*: Learning is facilitated when the instruction is designed to encourage integration into the real world [17]. The instructional design is capped with NPTs demonstrating and sharing their learning with a teaching experience that can be transferred to both OMS-1 students during the ultrasound lab and their future medical practice.

### Training and Teaching Lab Processes

Following training, the NPTs provided teaching support to OMS-1 students in two ultrasound labs using ultrasound imaging to reinforce normal anatomy. All OMS-1 students (*n* =  ~ 200) participate in two 1-h live standardized patient scanning sessions focusing on regional anatomy taught in conjunction with other components of the HAL course. The OMS-1 students are divided into four groups of around 50 students. Each group is assigned a specific 1-h time slot for their ultrasound lab. During the lab session, a standardized patient acted as a model, and two to three NPTs supported the learning of OMS-1 student learners. At each treatment table, a dedicated hand-held ultrasound device is provided for the scanning.

Ultrasound lab sessions involve a brief presentation by a faculty member on the relevant topic, including patient position, ultrasound settings, and examples of imaging. During the scanning session of regional anatomy, OMS-1 student learners use the standardized patient as an imaging model. NPTs provide ultrasound support to teams by guiding them as they practice ultrasound techniques and imaging anatomy.

### Data Collection

The near-peer teachers (*n* = 10) participated in individual semi-structured interviews that were scheduled after the completion of the second ultrasound lab. The semi-structured interviews used a set of questions to guide the conversation but enable flexibility to explore other emerging topics (see Appendix). To ensure that the interview questions addressed the research questions, a group of experts reviewed them prior to administration. The semi-structured interviews were conducted in a private conference room and were audio-recorded for transcription. Interviews lasted between 24 and 42 min, with the average interview lasting 30 min.

The researcher/designer maintained a journal for every ultrasound lab reflecting upon the participation and interaction between NPTs and first-year medical students and documenting the integration of newly acquired ultrasound skills. A reflective journal allows for transparency in the qualitative research process by allowing the researcher to share thoughts, observations, and opinions formed during the study [21].

### Data Analysis

The audio recordings were organized, transcribed verbatim, and coded using a deductive analysis approach via NVivo 14 software [22]. A deductive approach involves creating an organizing framework for codes prior to reviewing the transcript. In this case, the approach was based on Merrill’s FPI. These priori codes included (1) preparation for teaching OMS-1 students in ultrasound lab and developing skills for future medical practice (*problem*); (2) support and guidance provided during the asynchronous training (*application*); (3) impact on future medical practice (*integration*); (4) effectiveness of *demonstrations* during asynchronous training; and (5) effectiveness of *application* activities during asynchronous training. The process of coding involved identifying patterns and recurring phrases within collected data [15]. The research team thoroughly reviewed all transcripts and investigated the larger ideas from the interviews. To enhance the reliability and minimize bias, two research team members coded the transcripts separately and any discrepancies were resolved through discussion or review by the research team members. From there, major concepts within the data were refined into themes. To maintain confidentiality, each participant was identified by an anonymous randomized numbering system, such as OMS2-01, meaning year 2 osteopathic medical student, with a randomized number assigned to each of the ten participants.

The reflective journal data analysis looked for manifest content, which is specific and objective to the observation [15]. The analysis of journal data from observed ultrasound labs involved identifying recurring themes.

### Trustworthiness

The researcher addressed trustworthiness by using the constructs of credibility, transferability, dependability, and confirmability [23]. Credibility was established in several aspects during the study, including (1) the use of triangulation in the collection of data through both semi-structured interviews and a reflective observational journal; (2) informing participants that the study is only interested in their personal experience and that there are no right or wrong answers during the interview; (3) participants could decline to answer a question if they did not feel comfortable, and they could withdraw from the study at any time; and (4) the participants were given the opportunity to member check the data for accuracy during the data collection process. The study’s transferability and ability to be generalized for a wider population were considered by providing a detailed description of the participants, laboratory environment, and data collection sessions. This helped the readers assess the research’s applicability in other contexts. Various aspects of the study, such as research design, implementation, intervention design, data collection, and an introspective evaluation, were clearly described to address dependability in the research study. Confirmability was accounted for with an audit trail, including a detailed report on the research process, methodology, participant selection, intervention design, and data collection and analysis techniques.

### Ethical Considerations

In order to conduct the research study, approval was obtained from the New Mexico State University Institutional Research Board (#IRB 2307070502), as well as a Site Letter for Research from the southwestern osteopathic medical school. Before starting the study, every participant received and signed an informed consent document. This document contained a written description of the study’s purpose, emphasizing that participation was entirely voluntary, the right of participants to withdraw at any point, and the assurance that all information collected during data collection remained confidential.

## Results

Near-peer teachers affirmed that the FPIs provided an effective framework for design in medical education. After being asked about the effectiveness and efficiency of asynchronous training for near-peer teaching in the ultrasound imaging lab, five main themes correlating with Merrill’s First Principles emerged (see Table 1).

**Table 1:** Merrill’s First Principles of Instruction and correlated themes

Merrill’s First Principles of Instruction	Themes
***Problem-Centered:*** Learning is promoted when learners acquire skill in the context of real-world problems	Importance of acquiring the skills to teach OMS-1 students in the ultrasound lab Acquiring skills for teaching/communicating ultrasound imaging in future medical practice
***Activation:*** Learning is promoted when learners activate existing knowledge and skill as a foundation for new skills	Activating prior knowledge from the previous academic year to acquire new skills for teaching ultrasound
***Demonstration:*** Learning is promoted when learners observe a demonstration of the skill observed	Video demonstrations and resources helped to prepare for teaching the skills and techniques of ultrasound
***Application:*** Learning is promoted when learners apply their newly acquired skill to solve problems	The newly acquired skills were applied to solve varied problems found in the teaching/communicating of ultrasound imaging in the online training, with feedback to help with the learning process
***Integration:*** Learning is promoted when learners reflect on, discuss, and defend their newly acquired skills	The NPTs integrated the newly acquired skills they had learned with the OMS-1 students they were teaching in the lab, and reflected on how it may be used in their future medical practice

### Theme 1: Real-World Problems—The Importance of Acquiring the Skills to Teach OMS-1 Students in the Ultrasound Lab and Acquiring Skills for Teaching/Communicating Ultrasound Imaging in Future Medical Practice

Learning is promoted when learners acquire skills in the context of real-world problems [17]. NPTs felt like they acquired skills that were pertinent to real-world situations, including teaching the OMS-1 students in the ultrasound lab, as well as learning ultrasound skills for use in future medical practice. NPTs offered:

I felt prepared from the training in my ability to come into the ultrasound lab and answer any questions from first-year medical students. And if someone was completely lost, I felt confident in my ability to start them off and help them understand ultrasound. (OMS2-02)

I’m very interested in OB, so it’s [the instruction is] applicable. That’s why it’s super important to me personally to be able to participate [as an NPT] because I feel like I definitely need that exposure. (OMS2-03)

### Theme 2: Activate Prior Knowledge—Activating Prior Knowledge from the Previous Academic Year to Acquire New Skills for Teaching Ultrasound

The NPTs utilized the online, asynchronous learning environment to expand upon their previous knowledge while acquiring new skills:

It [the instruction] helped me review information that I might not have seen in a while. I felt like knowing the stuff I did from last year, combined with the asynchronous training, I felt like I was able to help them [OMS-1 students] more. (OMS2-04)

After reviewing the asynchronous training, including the PowerPoints, slides, and practice questions, I realized that all the information was still available from last year. (OMS2-08)

### Theme 3: Demonstration—Video Demonstrations and Resources from the Online Modules Helped to Prepare for Teaching Ultrasound Skills and Techniques, but Some Felt that They Still Needed to Have Hands-On Experience for Training

Most NPTs found the video demonstrations and online resources helped NPTs in preparing for teaching ultrasound images in the lab:

The videos were very helpful for the demonstrations because, in theory, you may know what it [the ultrasound imaging] looks like, but it's different in practice. (OMS2-06)

Maybe adding ten minutes before the ultrasound [lab] to run through exactly what they need to know. I think having a little session right before the class where individuals can ask questions to make sure that we’re all on the same page [would help]. (OMS2-03)

### Theme 4: Application—The Newly Acquired Skills Were Applied to Solve Varied Problems Found in the Teaching/Communicating of Ultrasound Imaging in the Online Training, with Feedback to Help with the Learning Process

NPTs stated that they were able to use the online training problems with direct feedback to access their own comprehensive knowledge of ultrasound imaging. According to some NPTs, additional problems and questions would be beneficial to the learning process:

I think they [online training problems] helped me really gauge [if] I actually know this material. Do I understand the entirety of it? And if I didn’t because of the feedback, I would go back to the slides before teaching. (OMS2-02)

They’re [online training problems] fundamental to cement the knowledge that you already have. If you learn something and then test it, you can apply it. (OMS2-08)

### Theme 5: Integration—The NPTs Integrated the Newly Acquired Skills They Had Learned with the OMS-1 Students They Were Teaching in the Lab, and Reflected on How It May Be Used in Their Future Medical Practice.

The NPTs found the online asynchronous modules were beneficial in helping to integrate their newly acquired skills into teaching practice and to reflect on the way that ultrasound imaging will be used in their future medical practice:

I want to potentially be an emergency medicine physician. I know they do utilize ultrasound a lot in the E.R., so this gave me more experience and training with ultrasound. (OMS2-06)

I am able to translate what I learned [in the instruction] into practice, particularly during the lab sessions with the OMS-1 students. (OMS2-10)

### Reflective Journal

The researcher’s reflective journal documented the NPTs’ interactions with OMS-1 students, their integration of newly acquired skills, and reflections on the use of ultrasound in their future medical practices. The following excerpts were documented by the researcher/designer during the two separate ultrasound labs.

#### Lab 1

##### OMS2-01

The NPT discussed not only ultrasound skills and techniques but discussed how they felt ultrasound would *impact future medical practice, specifically in MSK and Emergency Medicine.* This seemed to put validity and a high-yield mentality into the minds of the OMS-1 students at that specific table.

##### OMS2-05

[The NPT] provided help with ultrasound techniques, anatomical relationships, and *future medical practice* uses. At certain tables, the NPT was *able to bring in some of the second-year content knowledge that is more advanced to build upon the basic concepts*. This was not done at every table, but at tables where the students were doing well.

##### OMS2-10

I overheard the NPT that MSK was not their thing but that they liked the challenge of working through content they were not comfortable with to help them as a future family physician.

#### Lab 2

##### OMS2-03

[The NPT] still seemed to lack confidence in their ultrasound skills and teaching, but they were much more engaged during this second lab.

##### OMS2-07

Brought in some clinical applications from the training module for using ultrasound to support their teaching.

##### OMS2-09

Good about bringing different explanations into the ultrasound lab, such as explaining the shoulder joint from the cadaver lab and what to look for when they go back in.

## Discussion

This study examines the impact of online, asynchronous training of near-peer teachers in a medical school’s ultrasound component of the human anatomy lab. Online training modules based on Merrill’s FPI principles were developed around real-world scenarios to facilitate learning [17].

The NPTs reported that the online asynchronous training helped them feel prepared to answer questions from first-year medical students and provide a solid foundation for their future medical careers. The training allowed the NPTs to review information from the previous academic year and build upon that foundation to acquire new knowledge enhancing their ability to help OMS-1 students. Video demonstrations and online resources helped prepare NPTs for teaching ultrasound skills, such as the identification of anatomical structures on ultrasound images and ultrasound techniques. However, some NPTs believed that video demonstrations alone were not enough, and that hands-on experience with teaching was also needed to create a more significant impact on learning. The provided online training problems provided direct feedback, helping NPTs evaluate their comprehensive knowledge of ultrasound imaging. Some NPTs suggested adding additional problems and questions to the learning process, as they helped them gauge their understanding of the material and cement their existing knowledge. The integration of the acquired skills allowed NPTs to be able to translate their knowledge into practice, particularly during lab sessions with OMS-1 students. During these labs, the NPTs had the opportunity to refine their communication skills by explaining ultrasound techniques to first-year students who had minimal experience, thereby preparing them for future clinical practice.

Using Merrill’s FPI had an impact on the effectiveness and efficiency of the near-peer teacher training of medical students. The NPTs found it beneficial to use real-world problems and scenarios as a focal point in their learning. According to Merrill [17], people learn more effectively when they solve problems and build knowledge than when they memorize information. During the interview, the NPTs discussed the impact of the training on their future teaching and explanation of ultrasound imaging with OMS-1 students in the lab, as well as its significance for medical practice. This was further reinforced in observations documented in lab settings. Some NPTs used examples of where ultrasound would be used in their future specialty or brought in clinical correlations that helped with the teaching process.

Merrill [17] stated that activating learners’ prior knowledge is essential to building a foundation for new knowledge. All NPTs completed HAL’s ultrasound training the prior year, which provided the foundation to learn new ultrasound and instruction techniques. The asynchronous training provided the NPTs with information that activated their prior knowledge. Merrill [17] believes that combining instruction with appropriate demonstration using specific situations is more effective for learning. During the interviews, it was found that some NPTs considered video demonstrations to be effective for teaching in the lab, while others preferred a hands-on approach. A potential solution for future NPT training could be a hybrid model that combines both video and hands-on approaches.

The online, asynchronous training utilized problems, such as patient positioning, questions, and identification, and provided feedback to the NPTs. Merrill [17] emphasized the importance of utilizing newly acquired knowledge and skills to carry out tasks in the learning process. The application problems in the ultrasound lab reinforced the knowledge and allowed the NPTs to reflect on their understanding of the content. The laboratory observations reinforced this idea. Some NPTs used module application examples during OMS-1 lab.

Merrill [17] stated that well-designed instruction should offer learners the chance to apply their newly acquired knowledge or skill to a task or problem. The learners should be encouraged to reflect on their newly acquired skills [17]. The data demonstrated NPTs effectively applied new knowledge by teaching ultrasound skills to OMS-1 students. Several NPTs were observed discussing ultrasound in their future medical specialties, and how they see themselves using it in practice.

## Conclusion

This research analyzes the effectiveness of asynchronous NPT training that is designed based on the First Principles of Instruction created by Merrill [17]. The findings indicate that the design framework was effective overall, with potential for further improvement through the integration of a hybrid instructional model. Such a model could combine virtual training with a brief hands-on component to address any questions and clarify the use of ultrasound equipment for NPTs.

The insights from this study offer valuable guidance for other medical institutions looking to develop similar NPT programs. This approach is particularly beneficial for institutions that face challenges in providing formal training for NPTs, who often have limited availability due to other academic responsibilities. However, the feasibility of implementing this instructional model depends on several factors, including available resources, institutional support, and scheduling flexibility. While the principles demonstrated here have broad applicability, their successful implementation in other settings will require careful consideration of these logistical challenges. Nonetheless, the framework could be adapted for use in other laboratory-based courses, such as gross anatomy or clinical skills training, where similar constraints on NPT time and availability exist. With appropriate adjustments, this model has the potential for widespread adoption across diverse educational contexts.

However, the study does have limitations. The small sample size, limited to medical students from a private southwestern medical school, may not fully represent the broader student population. Additionally, participants were self-selected, having volunteered for the ultrasound component of the human anatomy lab and applied for the NPT program. Another limitation is the reliance on self-reported data, which may introduce bias.

Future research should aim to expand the scope of these findings by including multi-campus studies and longitudinal designs to increase the sample size and improve the generalizability of results. Another promising area for future exploration is the evaluation of a hybrid instructional model, combining asynchronous training with hands-on practice, to assess its feasibility and effectiveness in NPT programs.

In conclusion, further research into hybrid instructional models, alongside ongoing refinement of NPT training strategies, could play a significant role in advancing medical education and improving training opportunities for near-peer teachers.
